# Transforming the future of health: building learning health systems across the globe

**DOI:** 10.1093/haschl/qxaf103

**Published:** 2025-05-21

**Authors:** Sandra Yankah, Robert Saunders, Mark Tykocinski, Claudia Salzberg, Jonathan Gonzalez-Smith, Rachel Bonesteel, Cameron Joyce, Charles Kahn, Mark McClellan, Eyal Zimlichman

**Affiliations:** Duke-Margolis Institute for Health Policy, Washington, DC 20004, United States; Duke-Margolis Institute for Health Policy, Washington, DC 20004, United States; Department of Pathology and Genomic Medicine, Sidney Kimmel Medical College, Thomas Jefferson University, Philadelphia, PA 19107, United States; Future of Health, Washington, DC 20001, United States; Duke-Margolis Institute for Health Policy, Washington, DC 20004, United States; Duke-Margolis Institute for Health Policy, Washington, DC 20004, United States; Duke-Margolis Institute for Health Policy, Washington, DC 20004, United States; Federation of American Hospitals, Washington, DC 20001, United States; Duke-Margolis Institute for Health Policy, Washington, DC 20004, United States; Sheba Medical Center, Ramat Gan 52621, Israel

**Keywords:** learning health systems, innovation, delivery reform, decision-making support

## Abstract

Health care has faced disruptions over the past 5 years, including a global pandemic, supply chain interruptions, workforce shifts, and the introduction of new artificial intelligence (AI) tools. Health care organizations continue to leverage the learning health system (LHS) concept to adapt to these challenges through iterative feedback loops. The Future of Health (FOH), an international community of over 50 senior health leaders that focuses on shared challenges across international health systems, collaborated with the Duke-Margolis Institute for Health Policy in a consensus-building process with FOH members to identify opportunities for action in an LHS. Key areas for action identified include opportunities to leverage data and AI to support clinical decision-making, steps to create an organizational culture of learning, and strategies to engage patients and caregivers, illustrated through case examples.

## Teaser

What can health care leaders do to accelerate learnings and scale evidence-based interventions? This commentary describes 3 key areas for action from international health care leaders.

## Introduction

Health care has faced massive disruptions in recent years, including a global pandemic, supply chain interruptions, workforce shifts^1^ and shortages, and the introduction of artificial intelligence (AI) tools. To quickly adapt, organizations can leverage the learning health system (LHS) concept,^2^ which integrates research into care delivery through iterative feedback loops.^3^ Increasing data accessibility and advancements in technology present new opportunities to accelerate learning.

Organizations struggle to scale evidence-based interventions generated through LHS efforts.^4^ One barrier is the complexity introduced by increases in data, treatments, diagnostics, and AI tools. Further, longstanding challenges limit data accessibility and integration.^5^ New strategies are needed to leverage learning systems more effectively, recognizing that technology alone cannot produce an LHS. This will require committed leadership, supportive culture,^6^ and modified workflows.

## Approach

Founded in 2018, the Future of Health (FOH)^7^ is an international community of over 50 senior health leaders,^8^ focusing on shared challenges across international health systems. This research, conducted in partnership with the Duke-Margolis Institute for Health Policy, draws from peer-reviewed and gray literature, external experts, and FOH member perspectives in virtual meetings, interviews, and an in-person summit. Members identified action areas where health systems are making strides in advancing LHS principles (Table 1), illustrated through case examples.

**Table 1.** Summary of recommended action areas with examples from international health leaders.

Action area	Opportunities to advance action
Advancing data-driven decision-making	Democratizing access and transparency to timely and actionable data Leveraging new AI tools for analyzing internal data and supporting clinical decision-making
Fostering organizational culture to promote learning	Promoting a supportive organizational culture through leadership commitments to learning Providing other organizational tools, such as empowering clinicians and staff to leverage data to continually improve their work
Engaging patients and caregivers	Enhancing communication strategies Launching diverse Advisory Groups and learning networks Focusing learning on care outcomes and activities meaningful to patients

Abbreviation: AI, artificial intelligence.

## Advancing data-driven decision-making in health care

Rapid growth in data, especially real-world data (RWD),^9^ and advanced analytic tools, including AI,^10^ present opportunities to improve efficiency, effectiveness, and equity in care delivery and allow for sophisticated LHSs. Real-world data are data related to health status and/or delivery of health care collected from a variety of sources (eg, data derived from electronic health records).^11^ However, data-driven decision-making is often hindered by organizational silos, limited actionable data, and access barriers for clinicians.

### Utilizing synthetic data for faster learning on the front lines

Producing data-driven insights requires staff to access and meaningfully engage with data. Synthetic data, free of identifying patient information but mimicking RWD, allow frontline teams to quickly address clinical, financial, and operational questions without compromising patient privacy or violating regulations.^12^ The value of synthetic data is that it allows teams to explore data quickly and expand access across users without delays due to privacy rules or data governance processes associated with use of RWD. This flexibility supports cross-organizational ability to work with data directly, which can accelerate iterative learning and innovation—key pillars of an LHS.^13^

Sheba Medical Center in Israel utilizes MDCLONE, a self-service data analytics tool that enables simplified access to synthetic health care data.^14^ MDCLONE supports the ADAMS (Ask, Discover, Act, Measure, Share) Center, which supports Sheba by leveraging various data sources and using technologies like Natural Language Processing to allow providers to answer complex questions quickly. The ADAMS Center couples clinical teams with project managers, statisticians, and other professionals to identify improvement opportunities. This approach led to changes in administration of an anesthesia-reversal agent in the operating room, with an estimated annual cost savings of 120 000 US dollars, without affecting clinical outcomes.

The Ottawa Hospital in Canada similarly deployed MDCLONE's synthetic data approach for patient encounters to study risk factors for stroke in patients with cancer.^15^ The study provided evidence on improving stroke care with new treatment considerations and a focus on high-risk, comorbid conditions. Using synthetic data produced results similar to original data, supporting its use in cerebrovascular and cancer research. The use of synthetic data allowed researchers to easily test a hypothesis, support data sharing without privacy breaches, and provide data access in a rapid and reliable way.

### Leveraging representative data to effectively implement AI tools

Artificial intelligence opens new ways to support learning by analyzing larger quantities of data than can be assessed by human analysts.^10^ For example, the ADAMS Center has started to implement AI tools to evaluate appropriate drug usage with data-derived rationales, examine computed tomography (CT) scan findings and clinical outcomes to reduce observational hospitalizations, and optimize frequency of therapeutic drug monitoring for mood-stabilizing drugs.^16^ Generative AI tools built using large-language models have evolved rapidly and may be useful for tasks such as identifying medical errors^17^ and administrative simplification. However, these tools are new and need refining^18^ before being widely implemented. To reach its full potential, AI requires access to representative health care data to test models. Future of Health members are participating in initiatives like the Coalition for Health AI^19^ to collaboratively develop best practices for applying such tools.

## Creating a supportive environment for learning: culture, leadership, and incentives

Learning health systems require a supportive culture that prioritizes learning, transparent communication around shared goals, and timely feedback. Leadership is key to empowering clinicians and staff to engage in learning and providing them with the data and time needed to improve and implement changes.

As part of a large-scale population health improvement initiative across Singapore, SingHealth has leveraged LHS principles in implementing novel initiatives. The Healthier Singapore^20^ initiative, launched by Singapore's Ministry of Health, focused on promoting preventive care and improving health through personalized plans, community programs, and subsidies for chronic care. The initiative tasked SingHealth and key^21^ partners^22^ with managing whole-health needs of their populations, expanding beyond traditional operations to link residents with community resources.

SingHealth used a multipronged approach to measure effectiveness and foster a culture of improvement, conducting ongoing studies with key stakeholders using the Organization for Economic Co-operation and Development evaluation criteria^23^ to assess and recommend improvements. They also led a policy ethnographic study^24^ to evaluate implementation, governance models, and leader perceptions, identifying challenges and necessary conditions for success.

## Engaging patients and focusing learning on meaningful outcomes

Engaging patients ensures that learning efforts meet patient needs and draw on patients’ experiences in care delivery. Engaging patients requires developing clear communication, trust, collaboration, and establishing relationships.

The Healthier Together Learning Health Networks,^25^ convened by the James M. Anderson Center for Health Systems Excellence at Cincinnati Children's Hospital Medical Center, illustrate patient centricity in learning systems. Healthier Together enables multisite clinical networks to share data, methods, and priority learning areas to provide more robust and generalizable evidence to improve pediatric care. Multisite networks are especially important as learning opportunities face limitations due to small patient numbers at individual sites. The networks foster collaborations among patients and families, clinicians and staff, scientists, and community members and have also accelerated evidence development, with nearly 600 teams in 300 pediatric care organizations globally in 2024.^26^ A key element of these networks is the intentionality in patient and family collaboration.

The Anderson Center engages families through face-to-face meetings and continuously generates momentum to connect through listserv and monthly calls or webinars.^27^ It also utilizes shared decision-making in evidence-based processes to ensure that patients and families can communicate views on treatment options and potential trade-offs. By systematically implementing quality-improvement science and fostering greater engagement with patients and families, networks have improved outcomes, including enhanced rates of remission and physical functioning, and reductions in premature births, serious safety events, and mortality rates.^26^ One network, the Advanced Cardiac Therapies Improving Outcomes Network, successfully engaged patients and caregivers in coproduction of first-of-their-kind, user-friendly education materials for pediatric heart failure and ventricular assist devices.^28^ Coproduction has been identified as a key element to iterative improvement in health care delivery.^29^ ImproveCareNow network, an LHS focused on advancing care for individuals with pediatric inflammatory bowel diseases, uses quality-improvement techniques and coproduction through its patient and family advisory council to develop novel patient-led resources,^30^ including an ostomy toolkit for patients and their families.^31^

Patients can serve as powerful advocates within care delivery transformation. The Ottawa Hospital undertook an initiative to enhance patient experience within a surgical department through a quality-improvement program. The program involved a 2-step process, beginning with input from employees in different departments to identify issues and propose solutions. For instance, a quality leader in the orthopedic department developed a program, with buy-in from surgeons, to ensure transparency in patients’ experiences. A patient focus group then provided feedback on key takeaways from the retreat so both patients and staff informed proposed changes. As a result of this initiative, Ottawa Hospital developed a list of strategies to improve patient experience (eg, improve pain management training for residents).^32^

Advisory councils provide another avenue to solicit patient engagement in learning approaches. Massachusetts General Hospital (Mass General) in the United States integrated Patient and Family Advisory Councils^33^ across its system. These councils help center initiatives on the needs of patients and families. When Mass General enhanced the rollout of their virtual-care services, they leveraged the council to improve it. After testing with patient groups, Mass General identified a gap between how providers and patients interpreted the “waiting room” experience. As a result, they re-designed the waiting message, reducing confusion and no-shows. Further, patients highlighted the need to improve the communication portal by providing clearer guidelines on what issues are appropriate for portal messages vs those requiring a visit.

## Summary and conclusion: building sustainable learning systems to improve outcomes

The LHS paradigm presents a dynamic and adaptable strategy that health care systems worldwide can leverage to confront the ever-evolving challenges facing the health sector. Examples highlighted underscore the potential for moving into a new era of health delivery, one that is far more responsive and patient-centered, with the need to advance data-driven decision-making in health care and a supportive organizational culture that emphasizes continuous improvement, and engage patients to ensure meaningful outcomes. By committing to these principles, health systems can encourage a more responsive, equitable, and resilient health system infrastructure where innovation thrives and patient needs are at the forefront of care delivery.

## Supplementary Material

qxaf103_Supplementary_Data
